# MCADS: Simultaneous Detection and Analysis of 18 Chest Radiographic Abnormalities Using Multi-Label Deep Learning †

**DOI:** 10.3390/diagnostics16040585

**Published:** 2026-02-15

**Authors:** Paulius Bundza, Justas Trinkūnas

**Affiliations:** Department of Information Systems, Faculty of Fundamental Sciences, Vilnius Gediminas Technical University, 10223 Vilnius, Lithuania; justas.trinkunas@vilniustech.lt

**Keywords:** chest radiography, deep learning, multi-label classification, TorchXRayVision, Grad-CAM, diagnostic imaging, convolutional neural networks (CNN), medical image analysis, automated diagnosis

## Abstract

**Background/Objectives**: Chest radiography remains a fundamental diagnostic tool for evaluating thoracic disease, yet its interpretation requires considerable time and specialized expertise. Worldwide shortages of trained radiologists can lead to lengthy turnaround times and delayed treatment. This study introduces the Multi-label Chest Abnormality Detection System (MCADS), a deep-learning-driven platform designed to automatically identify and interpret 18 distinct radiographic abnormalities to address these diagnostic challenges. **Methods**: MCADS integrates a pre-trained DenseNet121 convolutional neural network (via TorchXRayVision) to balance broad pathology coverage with rapid inference. Images are processed asynchronously on a central server to avoid the interruption of clinical workflows. To enhance transparency and clinician confidence, the system employs Gradient-weighted Class Activation Mapping (Grad-CAM) to overlay heatmaps pinpointing image regions most influential to each predicted abnormality. The system was evaluated using eight large, publicly available datasets. **Results**: When evaluated on diverse datasets, MCADS achieved high area-under-the-curve performance metrics across all 18 target conditions. The platform consistently produced accurate, multi-condition analyses in under thirty seconds per image, demonstrating both reliability and speed suitable for clinical environments. **Conclusions**: MCADS demonstrates the potential to accelerate chest X-ray interpretation by delivering fast, reliable, and explainable multi-abnormality screening. Its deployment could reduce radiologist workload and mitigate diagnostic delays, offering a pathway to improve patient care within data-driven healthcare environments.

## 1. Introduction

Chest radiography remains a high-volume, first-line modality for the triage and longitudinal monitoring of cardiothoracic disease [1]. Building on advances in digital image acquisition, interpretation workflows leverage expert availability and systematic case prioritization to support clinical decision-making [1]. These systems facilitate efficient time-to-decision periods, manageable cognitive loads, and consistent documentation across clinical settings, effectively aligning demand with radiologist capacity. Routine services are supported by the automated prioritization of abnormal studies, efficient resource allocation for screening tasks, and the integrated storage of predictions and visual explanations. Concurrently, evidence continues to accumulate that deep-learning-assisted workflows improve abnormality detection on chest X-rays, underscoring the value of decision support in dynamic environments [2]. Robust, calibrated systems further ensure reliable operation at scale, effectively managing multi-label settings and diverse class distributions [1,3].

The Multi-Label Chest Abnormality Detection System (MCADS) addresses these gaps through an integrated, explainable, and operationally efficient platform for automated chest X-ray analysis. While MCADS adopts pre-trained convolutional neural networks from TorchXRayVision (DenseNet-121) [4], the core contribution lies in the operational workflow bridging the model and the clinical environment. The underlying model treats pathologies as multi-label classification tasks where the output layer utilizes a Sigmoid activation function; this ensures that, while shared convolutional features capture latent correlations (e.g., consolidation often correlating with pneumonia), the final probability scores are generated independently to allow for non-exclusive multi-label diagnosis. The models leverage composite corpora drawn from widely used datasets such as ChestX-ray14 [5], CheXpert [6], and PadChest [7], enabling broad coverage of common radiographic findings [4,5,6,7]. Image uploads are processed asynchronously on a central server using task queues and thread-based fallbacks to maintain responsiveness under load while avoiding interference with interactive clinical workflows. Predictions, metadata, and visualizations are presented in a unified data model to support longitudinal review, auditability, and secondary analysis without dependence on external applications.

A distinguishing feature is an embedded case-level prioritization mechanism that stratifies studies into insignificant, moderate, and significant findings using the average of available abnormality probabilities. Operational thresholds—categorizing findings as “Insignificant” (<19%), “Moderate” (20–30%), and “Significant” (>31%)—are heuristic values derived from the probability distributions observed during the integration of TorchXRayVision models. These thresholds are not hard-coded; they are loaded from a configuration file (calibration.json), allowing individual sites to dynamically tune the sensitivity/specificity trade-off without modifying the source code. This yields a simple yet interpretable triage signal that can surface high-priority cases to radiologists and clinical teams. These elements align with recommendations from recent reviews of deep learning for chest radiography [1]. Out-of-distribution scoring is attached to prediction outputs to flag atypical inputs that may warrant cautious interpretation [1].

Explainability is integral to the system’s design. Gradient-weighted Class Activation Mapping produces class-specific heatmaps and overlays that highlight the image regions most influential for each predicted abnormality [8]. All artifacts are stored alongside model probabilities and metadata, enabling transparent review, quality assurance, and education. The approach aligns with best practices in explainable AI for clinical imaging and remains compatible with emerging domain-specific, self-supervised pretraining paradigms for chest X-rays [9].

MCADS is implemented as a Django-based web application with asynchronous execution, progress tracking, and explicit error handling. The architecture emphasizes portability and maintainability, with containerized deployment via Nginx and Gunicorn and a production-ready PostgreSQL backend for transactional durability, indexing, and concurrency under realistic loads. Configuration files govern calibration and thresholds to separate inference policy from code, while explicit model fields for each target abnormality and a dedicated prediction history preserve probabilities, visualization paths, model identifiers, and derived severity level for traceability.

By coupling multi-label detection with calibrated probabilities, case-level prioritization, and embedded explainability in a single, auditable system of record, MCADS directly addresses the operational challenges of contemporary radiography services [1,2]. The resulting workflow aims to shorten turnaround times by elevating likely abnormal studies, reduce repetitive screening burden through automated inference at scale, and standardize storage and communication of predictions and visual evidence within data-driven care pathways [1,2,4,5,6,7].

## 2. Materials and Methods

### 2.1. System Architecture

The Multi-Label Chest Abnormality Detection System (MCADS) is a containerized, production-oriented web application that integrates a modern Django 5.2 web tier (Django Software Foundation, Huntersville, NC, USA), an asynchronous processing tier for compute-intensive inference and visualization workloads, and a set of infrastructure services for persistence, messaging, and delivery. The HTTP stack is designed around ASGI to support efficient concurrency for request handling and long-running background orchestration. In containerized execution, the application server runs under Gunicorn (Benoit Chesneau, Paris, France) with an ASGI worker UvicornWorker (Encode OSS Ltd., Brighton, UK), and is typically fronted by an Nginx reverse proxy (F5, Inc., Seattle, WA, USA) for TLS termination, caching headers, and static asset delivery. A WSGI entrypoint is available for compatibility where required, but the default runtime uses ASGI.

User interaction is provided through server-rendered templates with Bootstrap 5 styling (Twitter, San Francisco, CA, USA) integrated via the Django extension for Bootstrap. Static files are collected and served efficiently with compression and manifest hashing. The presentation layer is internationalized with language negotiation and locale middleware, with English and Lithuanian currently enabled. The HTTP request pipeline is hardened with a Content Security Policy, clickjacking protection, and strict cookie attributes, and it includes custom middleware for request rate limiting and role-based access control. In production, secure headers are enabled, HSTS is configured, and reverse-proxy awareness is enforced.

Compute-heavy operations are offloaded to a dedicated asynchronous processing tier using Celery (Celery Project, San Francisco, CA, USA). A Redis service (Redis Ltd., Mountain View, CA, USA) functions as the message broker and result backend, and a separate scheduler is provided for periodic maintenance tasks. Worker concurrency is intentionally conservative to respect memory constraints on CPU-only deployments. For environments without the worker stack, the system degrades gracefully by executing tasks in a lightweight background thread within the web process for basic inference and interpretability; segmentation tasks are intended for the worker tier. Health checks and log files enable lightweight observability for web and worker containers.

The machine learning subsystem centers on pre-trained models and datasets provided by TorchXRayVision [4] (Mila, Montreal, QC, Canada). Image preprocessing follows the canonical chest X-ray normalization and geometry pipeline, including modality-aware normalization, center cropping, and resizing. Classification uses pre-trained convolutional networks exposed by the library, loaded lazily and cached in-process to avoid repeated initialization. The system does not have a dedicated “Normal” output neuron; instead, a “Normal” or “No Findings” status is a derived state, occurring only when the probabilities for all 18 target pathologies fall below the “Low” severity thresholds configured in the system. Model artifacts are cached on a shared volume to minimize cold-start latency and external downloads across processes and containers. To ensure deterministic and stable CPU inference on small servers, vectorized backends that may trigger heavy memory use are disabled and intra-op threading is constrained.

Out-of-distribution gating is performed by an autoencoder from the same library. A reconstruction error computed on the preprocessed input is compared against a configurable threshold to flag uncertain cases for expert review. Post hoc calibration is applied to classification outputs via per-label temperature scaling and decision thresholds. Calibration parameters are loaded from environment variables or a mounted JSON configuration, and both calibrated scores and derived binary decisions are attached to the result payload for downstream presentation and auditing.

Interpretability facilities include class-discriminative activation visualizations and pixel-level saliency maps. The system supports single-pathology explanations and combined overlays weighted by predicted probabilities above a configurable threshold, enhancing case review and quality assurance. Visual artifacts are generated at analysis time, persisted to media storage, and linked to the corresponding analysis record for subsequent retrieval in the user interface and via AJAX endpoints. Anatomical segmentation is additionally supported through a pre-trained model PSPNet (Chinese University of Hong Kong, Hong Kong, China) to produce multi-channel masks for key thoracic structures; combined overlays and individual masks are saved to facilitate localization and structured review.

Persistence consists of a relational database for users, analyses, and visualization metadata, while large artifacts such as input images and generated overlays are stored in the media filesystem. A Redis cache may be enabled for application-level caching; when unavailable, a local in-memory cache provides a development fallback. The deployment topology separates concerns into distinct containers for the web server, asynchronous worker, scheduler, database, cache/broker, and reverse proxy. Initialization sequences wait on dependent services, conduct migrations, collect static assets, and establish writable directories for logs, media, exports, and backups. Model caches are mounted into both the web and worker containers to eliminate redundant downloads.

End-to-end, the runtime flow accepts an image upload, persists the payload, and dispatches background analysis. The system utilizes pydicom (Darcy Mason, London, ON, Canada) to parse DICOM files primarily for pixel extraction and format conversion (DICOM to PNG). In the current architecture, sensitive DICOM metadata (e.g., Patient ID) is not automatically extracted or persisted from file tags; instead, necessary patient information is entered separately via the upload interface. For production deployment, the backend is designed to discard the original DICOM PII after conversion to ensure compliance. Once computation is completed, the calibrated predictions, OOD indicators, and interpretability artifacts are attached to the case record and rendered in the results view. A history ledger tracks prior analyses for longitudinal review, and users can star records for rapid retrieval. The architecture is cross-platform and optimized for CPU-only environments, with ASGI-first serving, conservative worker concurrency, model caching, and explicit backend tuning to maintain stability and responsiveness under tight memory budgets.

### 2.2. Explainability

**Figure 1:**
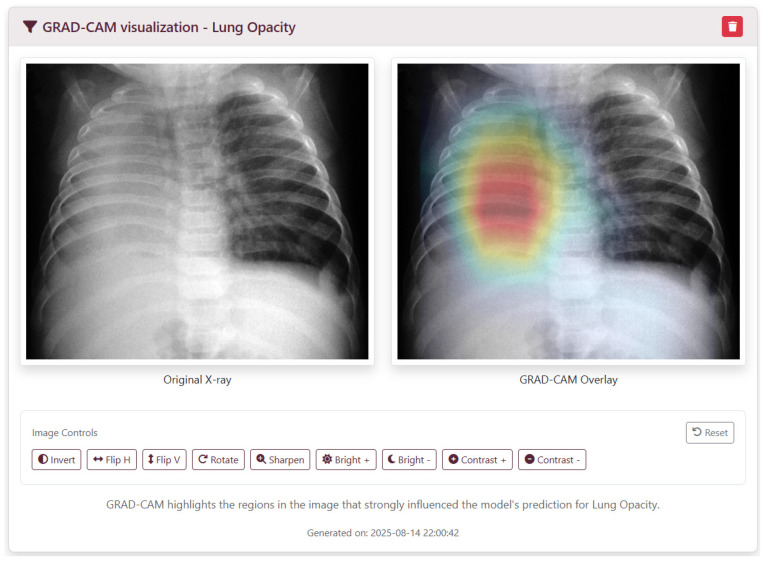
MCADS Explainability Interface (Grad-CAM). This view provides pixel-level attribution for a detected pathology (lung opacity), presenting a side-by-side comparison of the original X-ray and the GRAD-CAM Overlay. The superimposed heatmap identifies thoracic regions contributing most significantly to the model’s prediction (red indicating highest influence). The interface includes a suite of image controls (e.g., invert, sharpen, contrast) to facilitate detailed manual inspection of the radiographic findings [10].

Grad-CAM: Gradient-weighted Class Activation Mapping [8] computes class-specific localization maps by backpropagating gradients from the final convolutional layer. These heatmaps are overlaid on the original CXR to highlight regions that contribute most to each prediction (Figure 1).

### 2.3. Datasets and Evaluation Metrics

As MCADS utilizes the pre-trained densenet121-res224-all weights without retraining, we reference the rigorous benchmarks established in the original library publication to characterize the model’s expected performance. The dataset distributions and test splits presented in Table 1 and Table 2 are those defined and validated by the creators of the TorchXRayVision library, adhering to their official “held-out” test sets (20% split) to prevent data leakage. MCADS was evaluated on the following publicly available test sets.

### 2.4. Integration and User Interface

**Figure 2:**
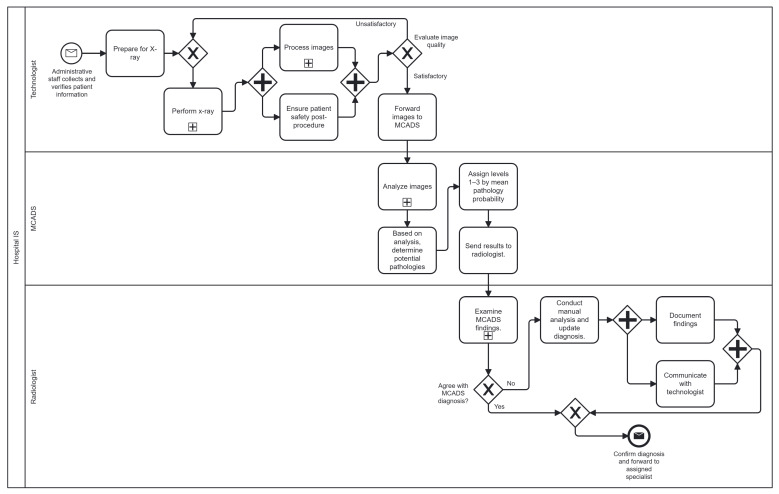
MCADS BPMN end-to-end process flow, from patient preparation and X-ray acquisition through automated analysis, radiologist review, and report forwarding.

(Figure 2) models the sequence of activities and decision points in MCADS. The Technologist lane begins with patient identification and preparation, followed by X-ray acquisition. Automated evaluation of image quality routes images either back for repeat acquisition if deemed unsatisfactory or forward to MCADS for processing if satisfactory. Within the MCADS lane, images undergo asynchronous preprocessing—normalization, center cropping, resizing—before multi-label inference and Grad-CAM heatmap generation. The resulting pathology probability scores and overlays are transmitted to the Radiologist lane, where examination of MCADS findings triggers either confirmation and forwarding of the report to the lung disease specialist or manual review, adjustment of diagnoses, and communication back to the technologist. This BPMN representation clarifies responsibilities, conditional flows, and integration points, guiding the implementation and validation of each process step.

**Figure 3:**
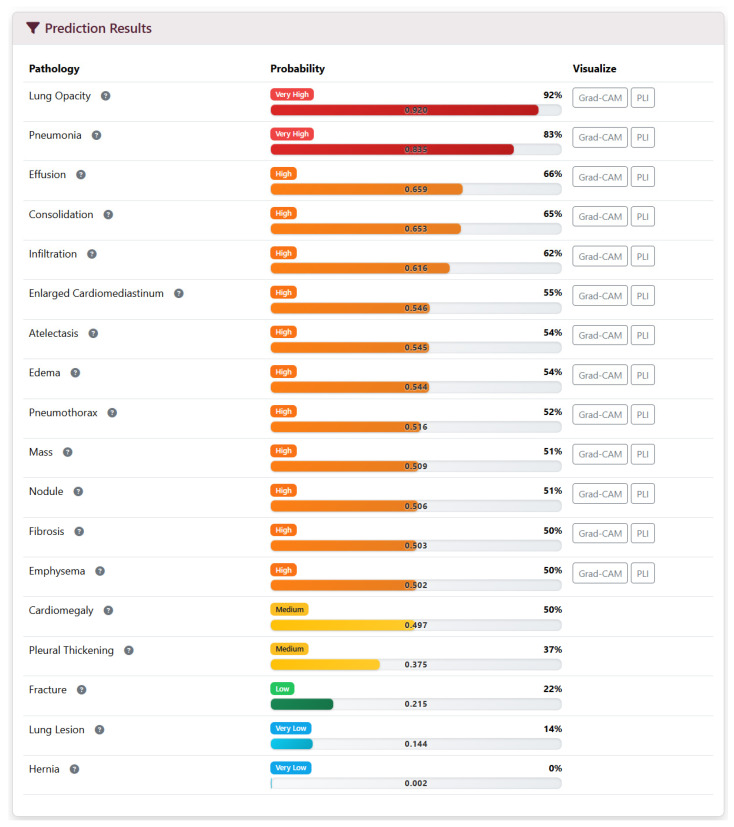
Bottom section of results page showing the probability scores for all 18 tracked pathologies. Bars are color-coded by severity threshold. Note. Model predictions and Grad-CAM findings were not certified or evaluated by radiologists.

**Figure 4:**
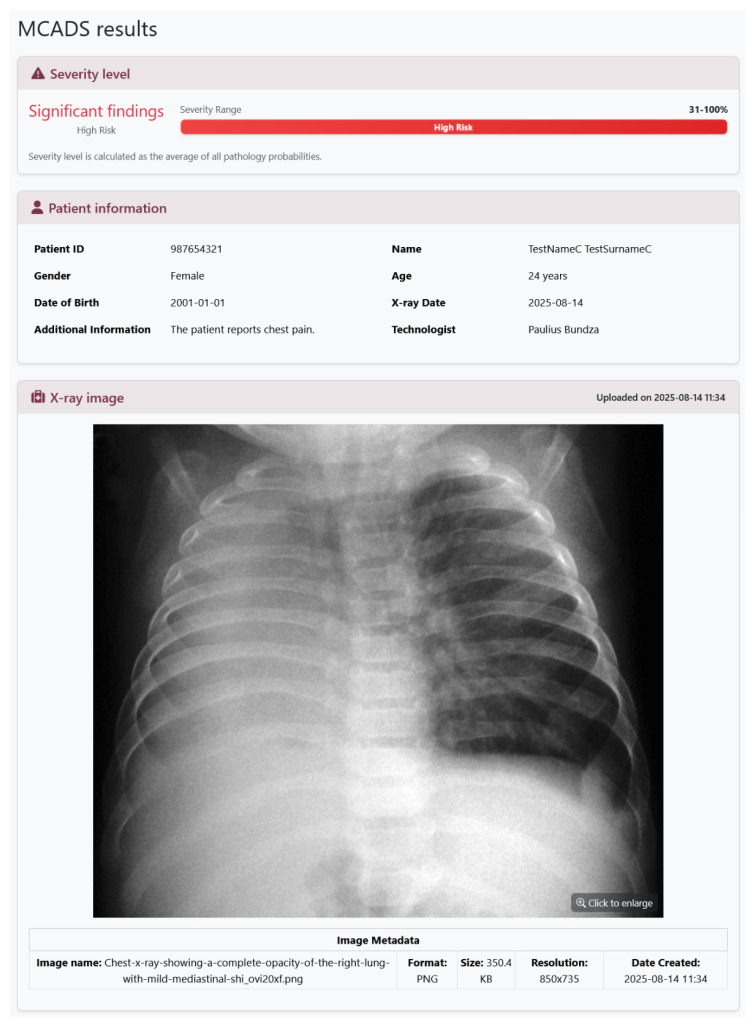
MCADS results interface (top section). This view presents the case-level summary, featuring: the Severity Level indicator; the Patient Information panel displaying session metadata; and the X-ray Image viewer for visual verification of the uploaded input file and its technical properties [10].

**Figure 5:**
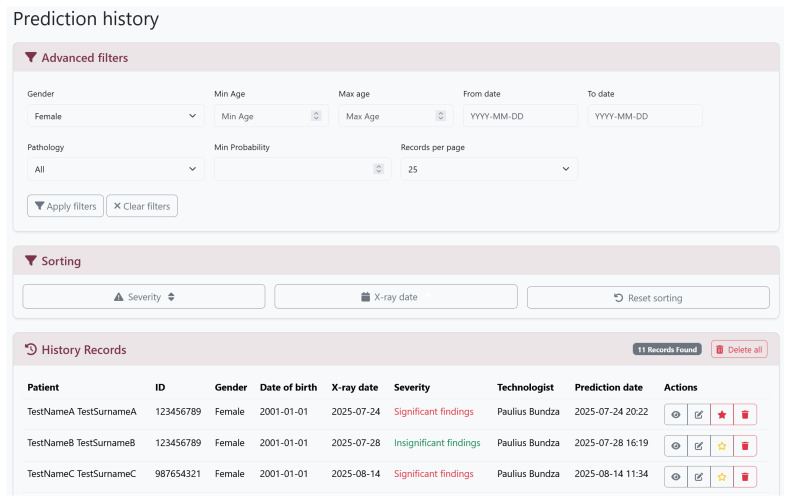
MCADS prediction history interface. This dashboard enables the longitudinal review of prior analyses through three key functional areas: the Advanced Filters panel for querying records by patient demographics, pathology type, or probability thresholds; the Sorting controls for organizing results by severity or acquisition date; and the History Records table, which lists processed cases with their derived Severity status (color-coded as “Significant”, “Moderate” or “Insignificant”) and provides quick-access actions to view, edit, or bookmark specific analyses.

The web interface was implemented using Django templates and Bootstrap 5. Clinicians can upload an image and view results in a single page, organized as follows:

1. Upload Panel: Input fields for patient metadata (ID, age, gender, clinical history) and file selection.
2. Loading Screen: Displayed while preprocessing and inference run, showing a progress indicator.
3. Results Panel: Includes Grad-CAM overlays positioned as thumbnails—clicking a thumbnail expands the visualization (Figure 1); probability scores for all 18 conditions (Figure 3); and a summary of detected pathologies (Figure 4).

4. History Tab: Lists prior analyses with filters by patient ID, date, and diagnosis (Figure 5).
5. Admin tab: Administrative and account-management functionalities are available via Django’s built-in admin interface.

## 3. Results

### Classification Performance

The DenseNet-121 model (“densenet121-res224-all”) consistently achieved high AUC-ROC values for most pathologies across all test datasets [4]. (Table 2) presents the AUC-ROC results for 18 pathologies. It is important to note that these values are point estimates cited directly from the comprehensive benchmarks provided by the library authors [4] to substantiate the selection of the DenseNet121 architecture; therefore, confidence intervals were not generated as part of this system-implementation study.

These results demonstrate that the model generalizes well across multiple data sources, even for less common pathologies such as fibrosis and hernia. No significant performance drop was observed when serving the model through the web application, indicating correct integration of preprocessing and inference pipelines [4].

## 4. Discussion

MCADS demonstrates that combining a pre-trained DenseNet-121 CNN with explainability techniques in a web-based application can achieve accurate, multi-label CXR analysis while maintaining transparency. We utilized DenseNet121 specifically because it offers an optimal balance between computational efficiency and accuracy for a web-based, CPU-optimized deployment. While Vision Transformers (ViT) may offer marginal performance gains, their computational overhead is higher, and DenseNet remains a standard benchmark for production-grade medical image analysis. Furthermore, unlike proprietary commercial systems where decision logic is opaque, MCADS offers a transparent architecture where probability calibration and interpretability layers are accessible and auditable [4]. Rare pathologies such as fibrosis and hernia—often underrepresented in individual datasets—achieved high detection performance due to multi-dataset pre-training.

Previously published studies of single-task models (e.g., pneumonia detection) have shown AUCs in the range of 0.83–0.91 [16,17]. Multi-label systems (e.g., ChestXNet, [18]) attained radiologist-level performance for a limited set of conditions. The comprehensive scope of MCADS, covering 18 pathologies, exceeds most prior works, which typically focus on 14 observations defined in ChestX-ray14 or CheXpert [5,6].

Integration of the Grad-CAM interpretability framework addresses clinician concerns about “black-box” models [8]. Heatmaps provide attribution that correlates with radiographic abnormalities, supporting trust in automated alerts.

MCADS’s implementation as a Django web application ensures accessibility: clinicians can upload images from any workstation without specialized software installations. Asynchronous processing prevents blocking of the user interface, and the database-backed history feature allows easy retrieval of prior analyses.

Limitations include the reliance on pre-trained models which, despite being trained on diverse corpora, remain vulnerable to domain shift; scanner-specific variations can cause performance drops. To mitigate this, the system includes an Autoencoder-based Out-of-Distribution (OOD) detection module to flag atypical inputs. Additionally, the current inference pipeline resizes images to 224 × 224 to match the classifier’s requirements. We acknowledge that this downsampling may result in the loss of fine-grained details necessary for detecting subtle pathologies (e.g., small nodules or hairline fractures). Future updates plan to support higher resolution inputs (512 × 512). Visual explanations (e.g., class-activation maps and gradient-based saliency) are post-hoc, sensitive to preprocessing and target selection, and can produce plausible yet misleading highlights; segmentation and overlays may be coarse and unsuitable for precise localization. Finally, the current study did not incorporate structured clinical information (e.g., radiology reports, lab values) that could further enhance predictive performance via multimodal fusion [19].

## 5. Conclusions

MCADS successfully integrates a pre-trained DenseNet-121 model with explainability mechanisms in a user-friendly, web-based platform to detect 18 chest radiographic abnormalities simultaneously. High AUC-ROC performance across 8 datasets confirms the model’s generalizability. Grad-CAM heatmap enhance interpretability, fostering clinician trust. Rapid inference (ranging from 5 to 30 s per image) positions MCADS as a promising second-reader tool. The variance in processing time is primarily due to the system’s caching mechanism: initial cold-start processing includes model loading, while subsequent inferences on the cached model (_model_cache) drop closer to the 5 s mark. Future research will focus on threshold optimization, prospective clinical validation and usability testing to validate the “Technologist to Radiologist” workflow, and the integration of multimodal data sources.

## Figures and Tables

**Table 1:** Used datasets with number of samples and classes [4].

Reference	Dataset	Number of Samples	Number of Classes
[5]	NIH ChestX-ray14	112,120	14
[6]	CheXpert	224,316	14
[11]	MIMIC-CXR (CheXpert labels)	377,110	14
[12]	Google (DS1)	759,611	4
[13]	RSNA Pneumonia Detection Challenge	30,000	2
[14]	SIIM-ACR Pneumothorax Segmentation	12,047	2
[7]	PadChest	160,000	16
[15]	VinBrain (VinDr-CXR)	18,000	6

**Table 2:** DenseNet-121 AUC-ROC values for 18 pathologies on held-out test sets (20% split) [4].

Dataset Pathology	NIH ChestX-ray14	Google	RSNA	SIIM	PadChest	VinBrain	CheXpert	MIMIC-CXR
Atelectasis	0.76	–	–	–	0.77	0.67	0.91	0.88
Cardiomegaly	0.88	–	–	–	0.93	0.90	0.91	0.88
Consolidation	0.77	–	–	–	0.88	0.93	0.90	0.91
Edema	0.85	–	–	–	0.97	–	0.92	0.92
Effusion	0.85	–	–	–	0.95	0.87	0.94	0.92
Emphysema	0.73	–	–	–	0.87	–	–	–
Fibrosis	0.72	–	–	–	0.94	–	–	–
Hernia	0.91	–	–	–	0.96	–	–	–
Infiltration	0.68	–	–	–	0.85	0.86	–	–
Mass	0.80	–	–	–	0.85	–	–	–
Nodule	0.69	–	–	–	0.85	–	–	–
Pleural Thickening	0.74	–	–	–	0.79	0.84	–	–
Pneumonia	0.71	–	0.86	–	0.82	–	0.84	0.82
Pneumothorax	0.75	0.85	–	0.79	0.81	0.93	0.85	0.81
Lung Opacity	–	0.92	0.88	–	0.87	0.85	0.87	0.86
Fracture	–	0.74	–	–	0.74	–	0.74	0.74
Enlarged Cardiomediastinum	–	–	–	–	–	–	0.78	0.84
Lung Lesion	–	–	–	–	–	–	0.84	0.82

Note. Dashes (–) indicate that the pathology label was unavailable in that dataset.

## Data Availability

The original data presented in the study are openly available in: Chest X-ray images used for UI demonstration can be reached here: https://www.kaggle.com/datasets/paultimothymooney/chest-xray-pneumonia. Original datasets as shown in Table 2 can be accessed here: RSNA Pneumonia Detection Challenge: https://www.kaggle.com/c/rsna-pneumonia-detection-challenge/data. CheXpert: https://aimi.stanford.edu/datasets/chexpert-chest-x-rays. NIH Chest X-ray8 (ChestX-ray14): https://nihcc.app.box.com/v/ChestXray-NIHCC. MIMIC-CXR: https://physionet.org/content/mimic-cxr/. PadChest: http://bimcv.cipf.es/bimcv-projects/padchest/. NIH Google Dataset: https://cloud.google.com/healthcare-api/docs/resources/public-datasets/nih-chest. SIIM-ACR Pneumothorax Segmentation: SIIM-ACR Pneumothorax Segmentation. VinDr-CXR: https://vindr.ai/cxr. All URLs were accessed on 13 January 2026.

## Abbreviations

The following abbreviations are used in this manuscript:

CNN	Convolutional Neural Network
Grad-CAM	Gradient-weighted Class Activation Mapping
CXR	Chest X-ray/Chest Radiography
AUC-ROC	Area Under the Curve—Receiver Operating Characteristic
OOD	Out-of-distribution
ASGI	Asynchronous Server Gateway Interface
WSGI	Web Server Gateway Interface
TLS	Transport Layer Security
HSTS	HTTP Strict Transport Security
AJAX	Asynchronous JavaScript and XML
JSON	JavaScript Object Notation
PSPNet	Pyramid Scene Parsing Network
